# Feasibility of dual-energy CBCT by spectral filtration of a dual-focus CNT x-ray source

**DOI:** 10.1371/journal.pone.0262713

**Published:** 2022-02-03

**Authors:** Boyuan Li, Derrek Spronk, Yueting Luo, Connor Puett, Christina R. Inscoe, Donald A. Tyndall, Yueh Z. Lee, Jianping Lu, Otto Zhou

**Affiliations:** 1 Department of Physics and Astronomy, University of North Carolina at Chapel Hill, Chapel Hill, North Carolina, United States of America; 2 Department of Applied Physical Sciences, University of North Carolina at Chapel Hill, Chapel Hill, North Carolina, United States of America; 3 Department of Biomedical Engineering, University of North Carolina at Chapel Hill, Chapel Hill, North Carolina, United States of America; 4 Adams School of Dentistry, University of North Carolina at Chapel Hill, Chapel Hill, North Carolina, United States of America; 5 Department of Radiology, University of North Carolina at Chapel Hill, Chapel Hill, North Carolina, United States of America; Virginia Commonwealth University, UNITED STATES

## Abstract

Cone beam computed tomography (CBCT) is now widely used in dentistry and growing areas of medical imaging. The presence of strong metal artifacts is however a major concern of using CBCT especially in dentistry due to the presence of highly attenuating dental restorations, fixed appliances, and implants. Virtual monoenergetic images (VMIs) synthesized from dual energy CT (DECT) datasets are known to reduce metal artifacts. Although several techniques exist for DECT imaging, they in general come with significantly increased equipment cost and not available in dental clinics. The objectives of this study were to investigate the feasibility of developing a low-cost dual energy CBCT (DE-CBCT) by retrofitting a regular CBCT scanner with a carbon nanotube (CNT) x-ray source with dual focal spots and corresponding low-energy (LE) and high-energy (HE) spectral filters. A testbed with a CNT field emission x-ray source (NuRay Technology, Chang Zhou, China), a flat panel detector (Teledyne, Waterloo, Canada), and a rotating object stage was used for this feasibility study. Two distinct polychromatic x-ray spectra with the mean photon energies of 66.7keV and 86.3keV were produced at a fixed 120kVp x-ray tube voltage by using Al+Au and Al+Sn foils as the respective LE and HE filters attached to the exist window of the x-ray source. The HE filter attenuated the x-ray photons more than the LE filter. The calculated post-object air kerma rate of the HE beam was 31.7% of the LE beam. An anthropomorphic head phantom (RANDO, Nuclear Associates, Hicksville, NY) with metal beads was imaged using the testbed and the images were reconstructed using an iterative volumetric CT reconstruction algorithm. The VMIs were synthesized using an image-domain basis materials decomposition method with energy ranging from 30 to 150keV. The results were compared to the reconstructed images from a single energy clinical dental CBCT scanner (CS9300, Carestream Dental, Atlanta, GA). A significant reduction of the metal artifacts was observed in the VMI images synthesized at high energies compared to those from the same object imaged by the clinical dental CBCT scanner. The ability of the CNT x-ray source to generate the output needed to compensate the reduction of photon flux due to attenuation from the spectral filters and to maintain the CT imaging time was evaluated. The results demonstrated the feasibility of DE-CBCT imaging using the proposed approach. Metal artifact reduction was achieved in VMIs synthesized. The x-ray output needed for the proposed DE-CBCT can be generated by a fixed-anode CNT x-ray source.

## Introduction

Since it was first approved by the U.S. Food and Drug Administration (FDA) twenty years ago, cone beam computed tomography (CBCT) has found widespread applications in dentistry, including dental implant planning and pre-operative and post-operative assessment of surgical procedures [1–4]. CBCT is also increasingly used in medicine, including image-guided radiation therapy [5, 6], intraoperative surgical guidance [7, 8], extremity imaging [9], and the diagnosis of head and neck disorders [7]. Using a flat panel detector (FPD) and conical radiation geometry, an entire volumetric dataset can be acquired with CBCT in a single rotation of the gantry, eliminating the need for patient translation. Compared to fan-beam CT, CBCT reduces the radiation exposure to the patient, cost, device footprint, and increases the spatial resolution [1, 2, 10].

However, the presence of strong metal artifacts has been a major limitation in using CBCT for dental imaging [11]. The strong x-ray attenuation from common metal features, such as dental restorations and implants, results in beam hardening and photon starvation. This leads to artifacts in the form of streaks and halos in the reconstructed 3D images [1, 11]. These artifacts degrade the image quality, compromise the diagnostic accuracy, and make dental CBCT ineffective for postoperative evaluation of the osseointegration of implants [12]. Various postprocessing techniques have been investigated for metal artifact reduction (MAR). However, the results of these algorithms have demonstrated limited utility [13].

Metal artifacts are typically less significant at higher energies, as better photon penetration is achieved. Virtual monoenergetic images (VMI) synthesized using dual-energy CT (DECT) datasets at high virtual monoenergetic energies were reported to reduce metal artifacts, particularly for small metallic objects such as dental implants [14, 15]. For example, Hokamp et al. reported reduction of metal artifacts caused by dental implants in VMIs using a helical CT with an energy sensitive detector [16]. The hyperdense artifacts were decreased significantly in VMIs at the virtual monoenergetic energies above 100 keV [16]. Today, DECT is typically performed using a fan-beam CT scanner with either dual x-ray sources operating at different anode voltages and two detectors, a single x-ray source with fast kVp switching, or an energy sensitive detector [17]. However, these CT systems are costly and are not typically available in dental clinics.

A DECT scanner using a single x-ray source and split spectral filters has also been introduced. It separates the polychromatic x-ray photons into two adjacent beams with distinct energy spectra [18, 19]. Compared to other methods for dual-energy imaging, the spectral filter approach has the advantage of a lower equipment cost but the disadvantages of reduced energy separation and decreased dose efficiency [18, 20]. The single-source spectral filtration method has also been applied to dual-energy CBCT (DE-CBCT) [21–23]. Iramina et al. investigated an “alternative spectral switching” approach by alternatively inserting Tungsten (W) and Tin (Sn) filters for a simultaneous beam-splitting method for DE-CBCT in a bench-top study [22]. A similar alternating filtration approach for DE-CBCT imaging was also reported by Fang et al. in a feasibility study [23]. Zhu et al. reported a clinical dental imaging study using a commercial DE-CBCT scanner [21] that combines rapid kVp switching with a rotating copper filter wheel to generate the low and high energy x-ray spectra [24]. A significant reduction of the metal artifacts was observed in the VMIs of both phantoms and patients, as compared to the conventional single energy CBCT images [21].

**Fig 1:**
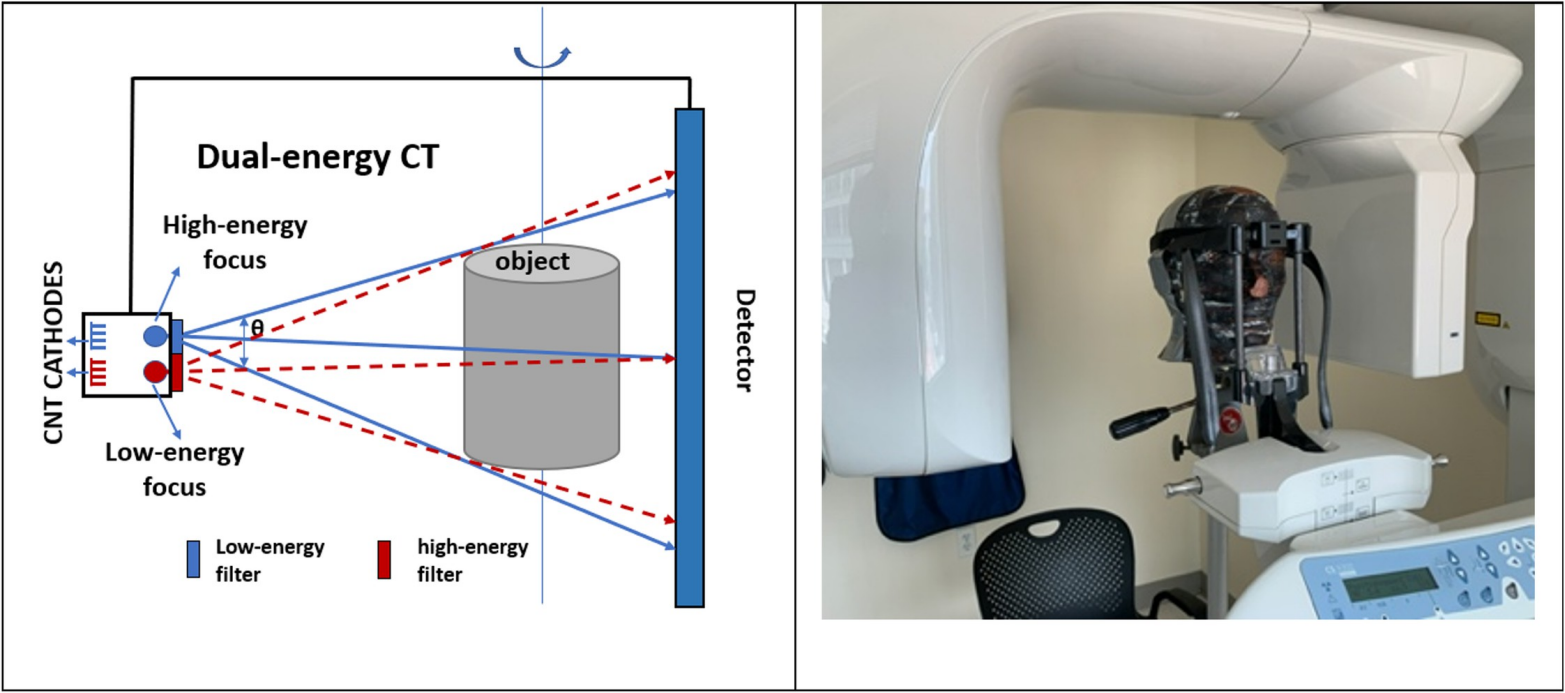
Drawing of proposed system and clinical scanner used for comparison. A drawing illustrating the proposed DE-CBCT using a CNT x-ray source with two focal spots and a common flat-panel detector (left). In the feasibility study the source and detector were stationary, and the object was rotated. For comparison the same head phantom was imaged using a clinical CBCT scanner (Carestream CS9300) (Right). A Rando adult skull and tissue-equivalent head phantom with a 1/4” and a 1/8” diameter stainless steel metal bead was imaged.

In this paper, we investigate the feasibility of developing a low-cost DE-CBCT without using either an energy sensitive detector or rapid high voltage switching, which significantly increases the equipment cost. The proposed approach is to retrofit a regular CBCT scanner with an x-ray source with dual focal spots which are filtered with respectively a low-energy (LE) and a high-energy (HE) spectral filter, as illustrated in Fig 1. The x-ray exposure alternates between the two beams to produce LE and HE projection images of the object, which are recorded on a common energy integrated detector. As the gantry rotates around the object, two complete sets of projection images are collected, one at LE and the other at HE, in a single gantry rotation. These projection image sets are then reconstructed into 3D volumes.

Although this setup could theoretically be achieved using an assembly of two separate conventional x-ray tubes, this application used a carbon nanotube (CNT) based x-ray source [25] with two independently controlled field emission cathodes with corresponding focal spots on a common W anode. This field emission source allows for rapid switching of the x-ray beams. Additionally, the x-ray flux and exposure time of each beam can be independently programmed in the CNT x-ray source [25, 26], allowing for optimization of the dose and noise levels in the LE and HE images.

In this study, the feasibility of the proposed method for DE-CBCT was investigated. X-ray energy spectrum simulation was performed to select the appropriate filters and to investigate the effect of spectral filtration on the x-ray tube output power. Projection images of an anthropomorphic head phantom were collected and reconstructed using an iterative reconstruction algorithm. VMIs were synthesized using the image domain basis materials decomposition method. The results were compared with the images of the same phantom from a clinical single-energy dental CBCT scanner.

## Materials and methods

### Experimental set-up

The experimental setup consisted of a CNT x-ray source, a detector, and a rotating object stage positioned on an optical table. The source and the detector were stationary and were controlled and synchronized by an electronic controller which also regulated the x-ray exposure. The phantom was rotated to collect the projection images for reconstruction in a step and shoot mode. The CNT x-ray source (NuRay Technology, Chang Zhou, China) has a W anode, a 1.0 IEC focal spot size, and a 0.4mm-thick stainless-steel x-ray window that serves both as a vacuum barrier and a filter. The source was operated at 120kVp. The detector (Teledyne, Waterloo, Canada) has an intrinsic 0.099 mm x 0.99 mm pixel size. The detector was 4x4 binned for this study.

The x-ray energy spectrum and the photon air kerma per current-time product (milliampere x second, or mAs) from the source were simulated for different spectral filters (materials and thicknesses) using the open-source Spektr software package from the Johns Hopkins University (www.istar.jhu.edu) [27]. It uses the Tungsten Anode Spectral Model using Interpolating Cubic Splines (TASMICS) [28] approach to approximate the photon fluence per tube mAs in 1 keV bins and the total air kerma rate per mAs.

A Rando adult skull and tissue-equivalent head phantom (Nuclear Associates, Hicksville, NY) was imaged. A 0.25-inch diameter stainless-steel metal bead was attached to the outer surface of the phantom and a 0.125-inch diameter stainless-steel bead was inserted to a socket near the teeth in the phantom to mimic the high atomic number materials commonly used for implants and dental restorations. CT imaging was performed in a step-and-shoot mode with the phantom rotating by 360 degrees in 1-degree step, in order to generate 360 projection images using the CNT x-ray source. Two separate scans were performed, first with a LE filter and then a HE filter, to experimentally simulate the proposed approach for DE-CBCT. For the LE CBCT scan, the x-ray source was operated at 120kVp, 19.2mA tube current, and 4ms exposure time per projection. The total x-ray exposure for the LE scan was 27.7mAs. For the HE CBCT scan, the source was operated at 120kVp, 24mA tube current, and 4ms exposure per projection. The total x-ray exposure for the HE scan was 34.6mAs. Multiple HE CBCT scans were repeated under the same conditions. For comparison, the same Rando head phantom was imaged using a clinical dental CBCT scanner (Carestream CS9300, Atlanta, GA) in the large field-of-view (FOV) mode (17x13.5cm). The manufacture default exposure condition was 90kVp, 5mA tube current, and 11.258 second total exposure time.

### Image reconstruction

The projection images were reconstructed using a model-based iterative reconstruction (IR) algorithm based on the open-source AIR Tools II and the ASTRA Toolbox [29, 30]. The reconstruction process is implemented in the MATLAB R2020b programming environment on a PC with Intel Core i5-9600K CPU @ 3.70GHz and NVIDIA GeForce RTX 2070 graphic card. The LE and HE projection images were reconstructed separately after dark and gain corrections.

### Synthesis of virtual monoenergetic image (VMI)

Because the proposed approach for DE-CBCT produces the LE and HE projections from two different physical locations with non-coincident x-ray beam paths, the image-domain basis material decomposition method was adapted to synthesize the VMIs [14]. The linear attenuation coefficients *μ*(*E*) derived from the reconstructed CBCT images at the LE and HE were expressed as the linear combination of the attenuations from the two basis materials as:

$$\mu \left(LE\right)=\tilde{\mu_{1}}\left(LE\right)\rho_{1}+\tilde{\mu_{2}}\left(LE\right)\rho_{2}$$

$$\mu \left(HE\right)=\tilde{\mu_{1}}\left(HE\right)\rho_{1}+\tilde{\mu_{2}}\left(HE\right)\rho_{2}$$

where $\tilde{\mu_{1,2}}\left(E\right)$ and *ρ*_1,2_ are the mass attenuation coefficients and densities of the two basis materials, respectively. In this study, water and iodine were chosen as the two basis materials. The *μ*(*LE*) and *μ*(*HE*) values were obtained from the reconstructed LE and HE images. The energy dependent mass attenuation coefficients were approximated at the mean energies of the LE and HE spectra. Using this information, the densities were computed for every voxel. The attenuation coefficient of the object at any monoenergetic energy, E, was then calculated by the linear combination:

$$\mu \left(E\right)=\tilde{\mu_{1}}\left(E\right)\rho_{1}+\tilde{\mu_{2}}\left(E\right)\rho_{2}$$

Where $\tilde{\mu_{1}}\left(E\right)$ *and* $\tilde{\mu_{2}}\left(E\right)$ are the mass attenuation coefficients at a particular monoenergetic energy, which were obtained from the X-Ray Mass Attenuation Coefficient database maintained by the National Institute of Science and Technology (NIST).

### Image analysis

The metal artifact index (MAI), which has previously been used as an indicator of the image distortion caused by the metal artifacts [21], was calculated at different monoenergetic energies (keV) as:

$$MAI=\sqrt{SD_{metal}^{2}-SD_{ref}^{2}}$$

where SD_metal_ is the standard deviation measured from an ROI placed in the soft tissue region where metal artifacts are present and SD_ref_ is the standard deviation measured from an ROI placed in the soft tissue region without metal artifacts in the same CBCT image. The contrast-to-noise ratio (CNR) was calculated using the Hounsfield Units (HU) in the reconstructed image:

$$CNR=\left|HU_{teeth}-HU_{tissue}\right|/SD_{tissue}$$

where HU_teeth_ is the averaged HU value within an ROI inside a tooth and HU_tissue_ is the HU value of an ROI in the soft tissue. SD_tissue_ is the standard deviation within the soft tissue ROI.

### Estimation of the x-ray exposure requirements for DE-CBCT

The x-ray exposure required was evaluated by assuming the total post-object air kerma of a DE-CBCT scan to be the same as a single-energy clinical dental CBCT scan. Furthermore, the total air kerma was assumed to be allocated equally between the LE and HE scans, which is preferred in dual energy imaging [15]. The x-ray exposure conditions used in dental CBCT scanners from different vendors and different imaging protocols vary significantly [31]. Here the Carestream CS9300 model in the large FOV mode was used as the reference. Its total filtration was listed as 2.5mm Al+0.1mm Cu. The total air kerma was calculated by multiplying the calculated air kerma rate with the total x-ray exposure (mAs).

The DE-CBCT imaging time (ΔT_scan_) and the x-ray exposure time per view (Δt_exp_) were calculated by:

$$\Delta T_{scan}=N_{view}\;x\;(\Delta t_{exp}\left(LE\right)+\Delta t_{readout}+\Delta t_{exp}\left(HE\right)+\Delta t_{readout})$$

$$\Delta t_{exp}\left(E\right)=mAs\;\left(E\right)/\left(N_{view}x\;I_{tube}\left(E\right)\right)$$

where N_view_ is the number of projection views and Δt_exp_(E) (E = LE, HE) the x-ray exposure time per view for each x-ray spectrum, Δt_readout_ the FPD readout time per frame, I_tube_(E) the x-ray tube current in mA, and mAs(E) the x-ray source exposure in mA*second.

## Results

### Simulated spectral filtration

**Fig 2:**
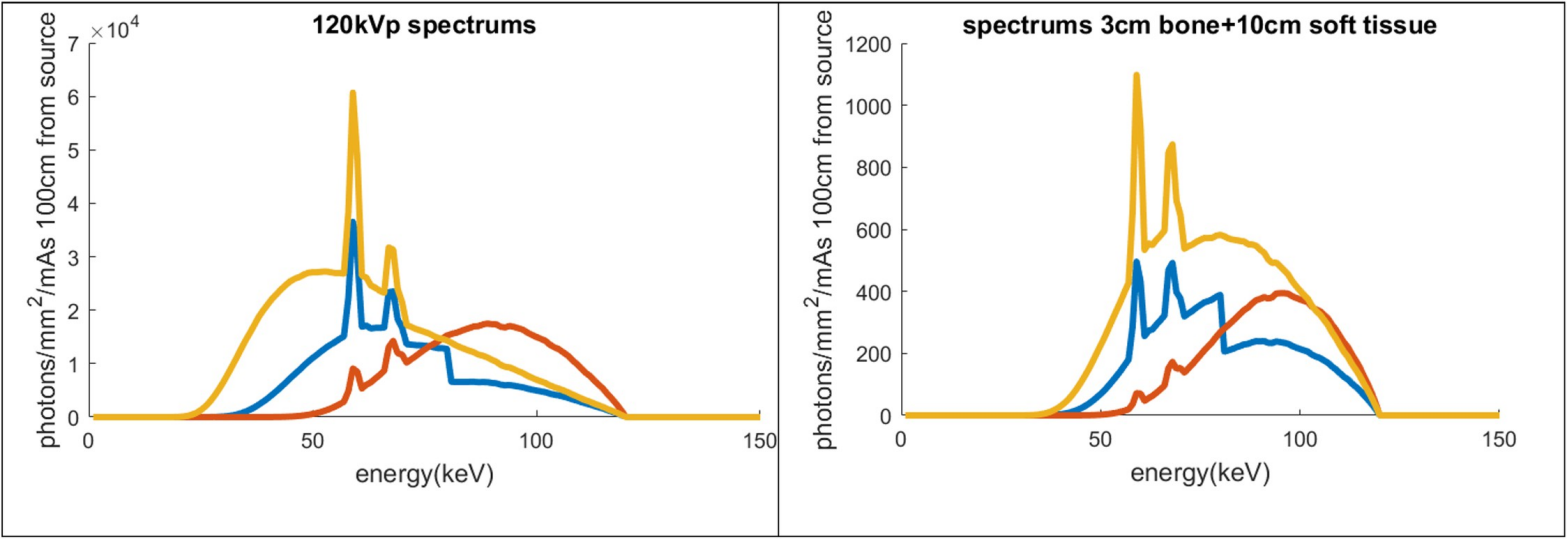
The simulated x-ray energy spectra. The simulated x-ray energy spectra from an x-ray source with W anode, 120kVp tube voltage before (left) and after (right) the object. Yellow: with the intrinsic filter (0.4mm Fe); Blue: with the low-energy filter (7mmAl + 0.05mm Au); and Red: with the high-energy filter (11mm Al + 0.63mm Sn). A multiplication factor was applied so that the low-energy and high-energy spectra combined produces approximately the same air kerma (per mAs) as the original spectrum.

Based on the x-ray energy spectral simulations, a filter containing 7mm Aluminum (Al) plus 0.05mm Gold (Au) was chosen as the LE filter, and a filter containing 11mm Al plus 0.63mm Tin (Sn) was selected as the HE filter, for the x-ray source operating at 120kVp. The resulting spectra are shown in Fig 2. These filters were selected based on their x-ray absorption K-edge energy levels. The filter thicknesses were selected to balance the amount of filtration achieved with the overall attenuation of the x-ray beam. Au has a K-edge at 80.7 keV, which is effective in attenuating the high energy x-ray photons, while Sn, with a K-edge at 29.2 keV, attenuates the lower energy x-ray photons. These filters are similar to those used in the split-filter helical CT scanner [18]. The calculated mean energies of the LE and HE spectra pre-object are 66.6 keV and 85.6 keV, respectively, at 120kVp. The HE filter attenuates the x-ray photons significantly more than the LE filter. The calculated air kerma rate (per mAs of source exposure) of the HE beam is 18.7% of the LE beam (Table 1). The object, simulated by 3cm of bone and 10cm of soft tissue, hardens the beam further by attenuating the low-energy photons more than the high energy photons. The air kerma rate of the HE beam behind the object becomes 31.7% of the LE beam.

**Table 1:** The calculated imaging parameters of the CNT x-ray DE-CBCT at 120kVp at 15mA and the specifications of a commercial CBCT scanner.

	Ref. CBCT	LE beam	HE beam
Tube voltage (kVp)	90	120	120
Tube current (mA)	5^a^^)^	15	15
Filtration	2.5mm Al +0.1mm Cu	7mm Al + 0.05mm Au	11mm Al + 0.63mm Sn
Mean energy (keV) (pre-object)	50.9	66.7	86.3
Air Kerma rate pre-object (mGy/mAs)^b^^)^	4.11x10^-2^	2.45x10^-2^	4.58x10^-3^
Air Kerma rate post-object^b^^)^, (mGy/mAs)	3.77x10^-4^	6.33x10^-4^	2.13x10^-4^
x-ray exposure (mAs)	56.3	16.8	49.8
Exposure time per view (ms)		3.1^c^^)^	9.2^c^^)^
Total exposure time (second)	11.258^a^^)^	18.8^d^^)^	

a) Information obtained from the DICOM image file.

b) From simulation.

c) Simulated value using 3cm bone + 10cm soft tissue.

d) Assuming 15mA tube current.

### Experimental CT images

**Fig 3:**
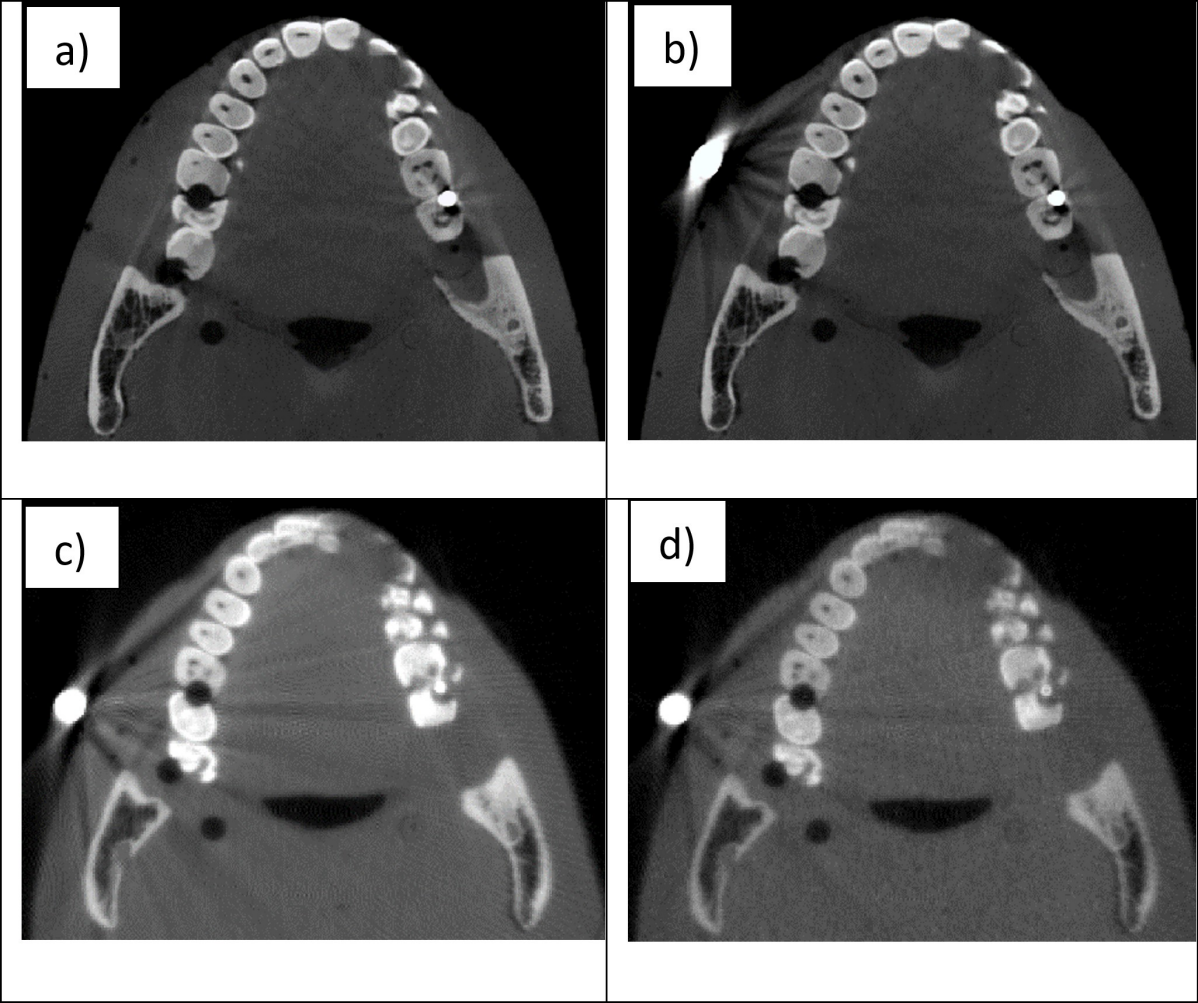
Reconstructed CBCT images of the Rando phantom. a) the clinical CBCT scanner without the external metal bead; b) the clinical scanner after attaching the external metal bead; c) the testbed using the LE beam; and d) the testbed using the HE beam. Because of the slight differences in the phantom positioning, the images from the clinical scanner and the testbed are not at same plane.

Fig 3 shows an axial slice of the reconstructed CBCT image of the Rando phantom before (Fig 3A) and after (Fig 3B) attaching a metal bead from the clinical CBCT scanner. The CBCT reconstruction was performed using the software package from the vendor. No additional post processing was applied. The presence of the metal beads, especially the larger bead, produces prominent dark streaks radiating out from the bead in the CBCT image (Fig 3B). In actual patient imaging, these artifacts may obscure lesions and degrade the diagnostic accuracy. The LE and HE CBCT images of the same phantom imaged using the testbed CNT device and reconstructed using the model-based iterative reconstruction algorithm are shown in Fig 3C and 3D. The overall qualities are comparable to that from the clinical scanner. However, the noise level of the HE image is noticeably higher than that in the LE image. This is attributed to the lower imaging dose caused by the strong attenuation from the HE filter, as discussed above. To reduce the noise level, four sets of HE projection images collected under the same conditions were averaged prior to reconstruction.

### Virtual monoenergetic images

**Fig 4:**
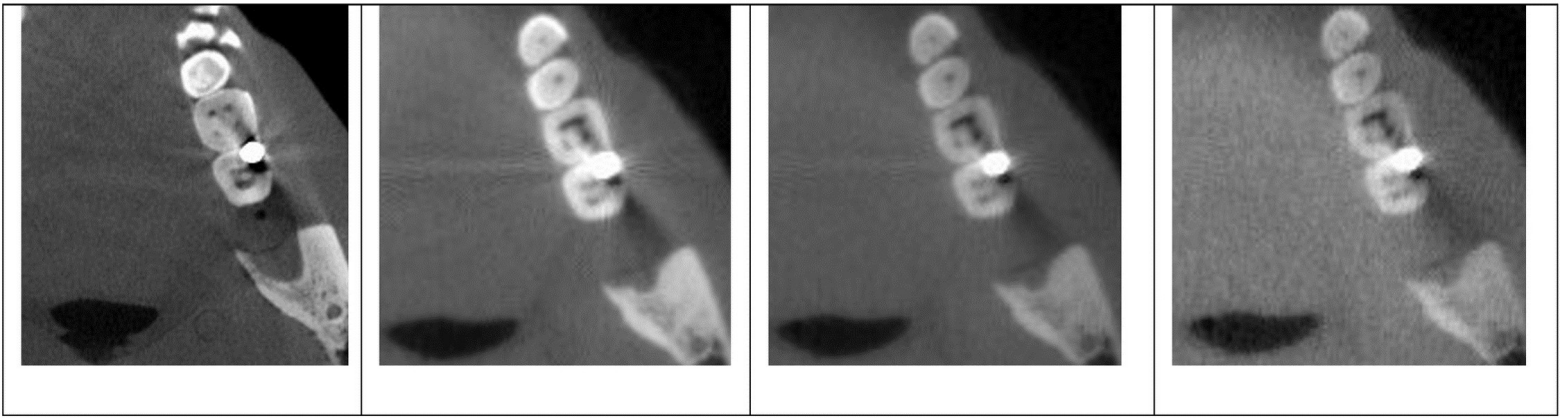
Zoomed in VMIs focusing on the region with the metal artifact. From left to right: 40, 60, 80 KeV (1^st^ row); and 100, 120 and 140keV (2^nd^ row). The images were set at the same window level (750HU:1500HU).

The virtual monoenergetic images were synthesized within the energy range of 40–140 keV using the image-based decomposition method. The inputs were the LE and the averaged HE images, which were processed in MATLAB. Fig 4 shows the synthesized images around the region with the large metal bead. The effectiveness of MAR depends on the virtual monoenergetic energy and is more effective at higher energies than lower energies. The dark streaks caused by the strong attenuation from the metal bead are significantly diminished in the VMIs at 120 and 140 keV compared to single energy CBCT images. This is attributed to the reduction of photon starvation at high energies and is consistent with the previous reports [16]. The metal artifacts are however not completely removed.

**Fig 5:**
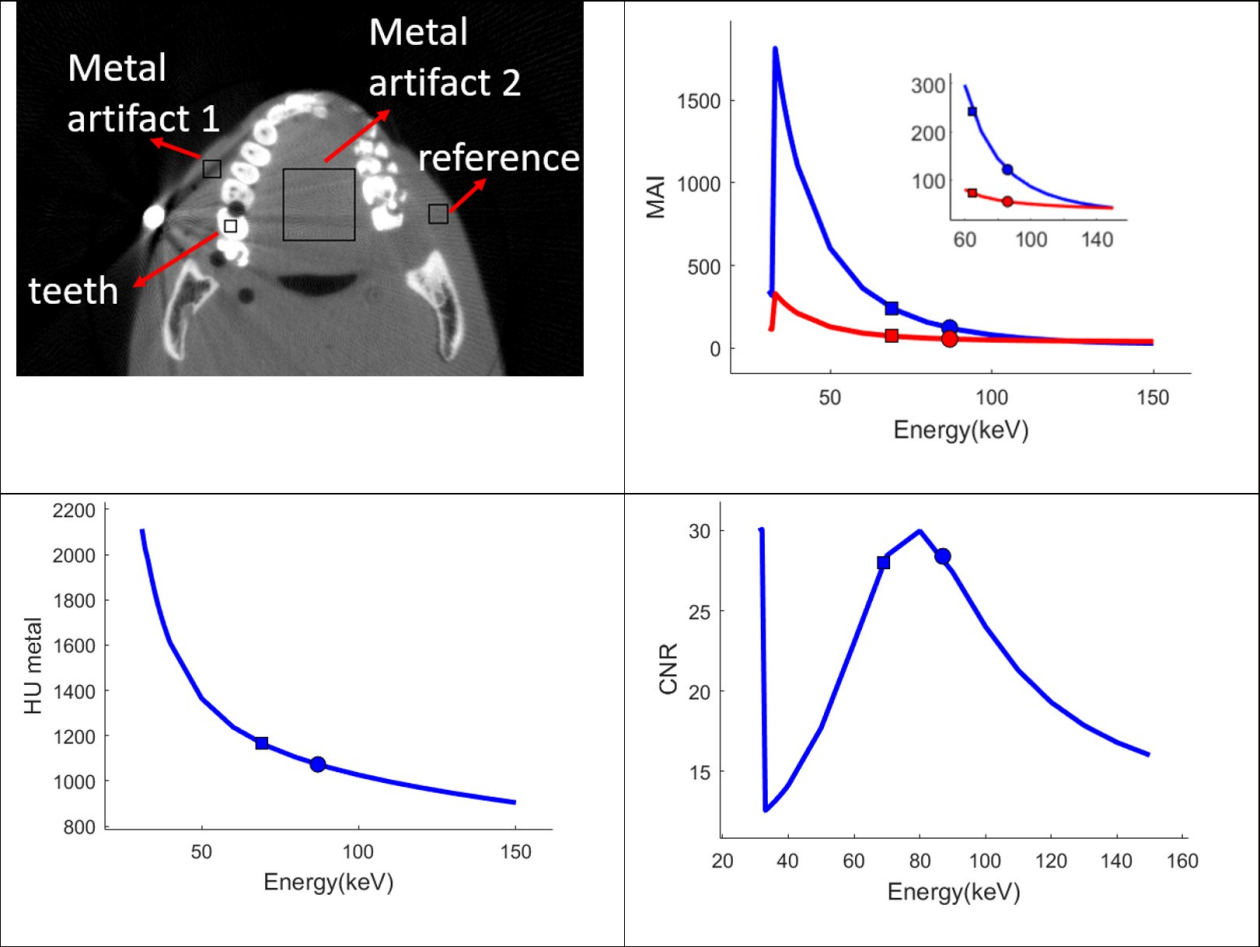
ROI and quantitative analysis. a) A CT image of the phantom showing the region-of-interest (ROI) used for the calculation. b), c), and d): The metal artifact index (MAI), Hounsfield Unit (HU) values as a function of the monoenergetic energy. The square and circle correspond to the values from the input low and high energy images respectively. In the MAI plot, the blue line is from the metal artifact region 1, and the red is from the metal artifact region 2 indicated in a).

The metal artifact index (MAI) was calculated for the two ROIs indicated in Fig 5. For comparison, the values derived from the reconstructed LE and HE images were also computed. Between 40–150 keV, the MAI value decreases with increasing energy, consistent with the visual observation of reduction of the metal artifacts. The lowest MAI is achieved at 150keV, the highest energy used in the calculation. As expected from the method used to calculate the VMIs, the MAI values from the LE and HE images fall on the energy dependence curve at the respective mean energies. Between 30-40keV a sharp increase of the MAI is observed. A plot with a finer energy step size around this region shows the increase occurs around 33keV, which is the iodine K-edge absorption energy. The averaged HU value of an ROI containing a dark streak decreases from -1150HU at the mean energy of the LE spectrum to -950HU at 150keV, as shown in Fig 5C. The contrast to noise ratio (CNR) between an ROI in a tooth and an ROI in the soft tissue with minimum metal artifact is shown as a function of the monoenergetic energy in Fig 5D. The highest CNR is observed at 80keV, after which the CNR decreases with increasing energy. The initial rise of the CNR at the low energy is associated with the K-edge absorption of the iodine component.

**Fig 6:**
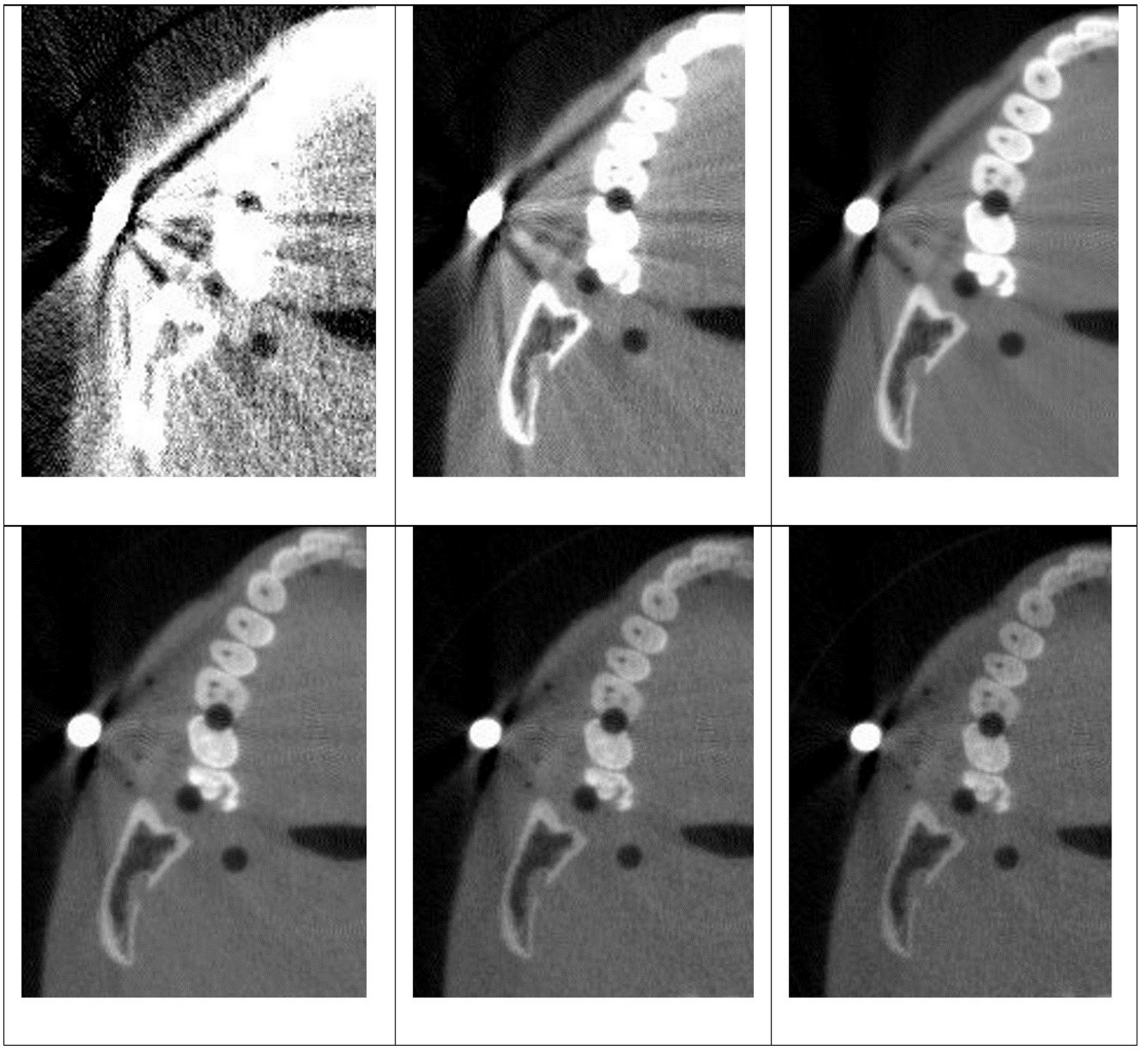
Region with the metal artifacts from the small metal bead. From left to right: Single-energy clinical CBCT, LE, HE, and VMI at 120KeV.

The metal artifacts caused by the small metal bead (0.125-inch diameter) are less severe compared to the large bead (0.25-inch diameter) at the energies used in this study. Fig 6 shows the reconstructed single energy CT images around the region with the small bead and the VMI at 120keV. The metal artifacts are more significant in the image collected using the LE spectrum than the HE spectrum. Additionally, these artifacts are reduced in the VMI at 120keV.

### X-ray output required and exposure time

Using the total air kerma of the clinical CBCT scanner as the reference, the required x-ray exposures for the LE and HE beams of the DE-CBCT scanner were determined using their respective calculated air kerma rates. The values are 16.8mAs and 49.8mAs for LE and HE beams respectively.

The total DE-CBCT scanning time ΔT_scan_ is estimated to be 18.8s using the above x-ray exposures and the assumption of N_view_ = 360, Δt_readout_ = 20msec/frame (50 frames per second detector), and I_tube_ = 15mA. The calculated total scanning time is comparable to that of a regular single energy spectrum dental CBCT scan, and the reported value of 30s for a DE-CBCT scanner with kVp switch and a rotating filter wheel [21]. The tube current of 15mA is well within the capability of a fixed-anode CNT x-ray source. In this study the CNT x-ray source was operated at either 19 or 24mA. The number of projection views (basis images) used in commercial dental CBCT scanners vary and some are not disclosed in the public literature [31]. The assumed value of 360 views in the estimate, which is the number of projections used in this experiment, is within the range commonly used [31].

The calculated exposure times per projection view are Δt_exp_(LE) = 3.1ms and Δt_exp_(HE) = 9.2ms. The increased exposure time Δt_exp_ for the HE beam increases image blur caused by the x-ray focal spot and detector motions compared to the LE beam. The amount of focal spot blurring is estimated to be 0.41mm/1.2mm for the LE/HE sources using the respective exposure level of 16.8mAs/49.8mAs, 15mA tube current, and a source-to-object distance (SOD) of 400mm.

## Discussion

The results demonstrated that the virtual monoenergetic images generated using the proposed DE-CBCT were effective in reducing the metal artifacts caused by the presence of metal objects in the anthropomorphic head phantom, consistent with prior reports using other dual-energy CT/CBCT techniques. For the filtration-based dual-energy imaging methods, a major question is whether a sufficient x-ray exposure can be delivered within an appropriate amount of time because the additional attenuation from the spectral filters significantly increases the x-ray output required. The results from this study show that, for the intended dental imaging applications, the required x-ray flux can be delivered by a fixed anode CNT x-ray source and the DE-CBCT scanning time is comparable to that of the current single-energy CBCT at the same imaging dose.

Because of the stronger attenuation by the HE filter, a longer exposure time per projection is needed for the HE image compared to the LE image at the same tube current to match the imaging dose at the two energy spectra. This can lead to a more severe image blur in the HE images compared to the LE images due to the x-ray source and detector motion during image acquisition.

The image blur caused by source and detector motions is not well documented for commercial dental CBCT scanners. One study reported a longer exposure time per projection in some commercial systems [31], suggesting a larger motion blur compared to the proposed DE-CBCT scanner. There are several options to reduce the motion blur. The present calculation was based on the assumption of 360° degrees gantry rotation. Reducing that to 180° degrees plus the fan angle would decrease the gantry rotation speed and the amount of motion blur by nearly 50% at the same imaging time. Increasing the x-ray tube current for the HE beam will reduce the exposure time. There is however a limit on the x-ray tube current at a given focal spot size due to the anode heat management. Most of the current commercial dental CBCT scanners use x-ray tubes with a static focal spot size of 0.5–0.7mm and operate at 5-15mA [32]. The CNT x-ray source used in this study has a slightly larger focal spot size of IEC 1.0 and was operated at 24mA @ 120kVp. Although a higher tube current was demonstrated in a separate study [33], the long term heat dissipation needs to be evaluated.

This feasibility study has several limitations. Partially due to concerns of the photon attenuation, the spectral filters selected produced ~20keV separation between the mean photon energies of the LE and HE beams at the fixed x-ray tube voltage of 120kVp. In addition, because the LE and the HE beams do not coincide in the proposed imaging geometry, an image-domain based decomposition method was used, which is known to be less effective than the project-domain based decomposition method. Under these conditions, although significant reduction was achieved, the metal artifacts were not completed removed. It would be desirable to include other common materials such as titanium and zirconia, in addition to the stainless-steel bead used for the imaging study. Because of the similarity in the atomic numbers, one expects that titanium will behave similar to the stainless-steel beads. On the other hand, stronger metal artifacts are expected from zirconia. These limitations however would not change the basic conclusions of this study.

The goal of this project is to develop a low-cost DE-CBCT for clinical 3D dental imaging. To further improve the effectiveness of metal artifact a more advanced algorithm [34, 35] will be adopted to perform materials decomposition and reconstruction in one step in a future study. Work is also under way to construct a fully functional prototype and to a systematic study to compare its performance with a conventional CBCT scanner.

## Conclusion

The results from this study demonstrated the feasibility of performing filter-based DE-CBCT imaging using an x-ray source with two focal spots operating at the same tube voltage. Metal artifact reduction was achieved in virtual monoenergetic images synthesized at higher energies. A The analysis showed the x-ray output needed for DE-CBCT can be generated using a fixed-anode CNT x-ray source.

## Supporting information

S1 Data (XLSX)

S1 Raw images (ZIP)
