# Supplementary figures and images for: Feasibility of dual-energy CBCT by spectral filtration of a dual-focus CNT x-ray source
Source: PLoS One. 2022 Feb 3;17(2):e0262713. doi: 10.1371/journal.pone.0262713 (PMC8812859; doi:10.1371/journal.pone.0262713)

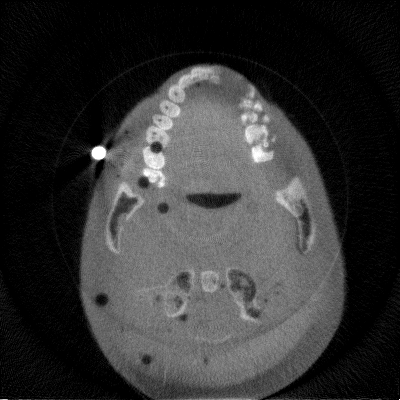

Supplement: S1 Raw images — (ZIP) [file pone.0262713.s002.zip › raw image data PLOS ONE/VMIs/image_VMI_150keV.tif]

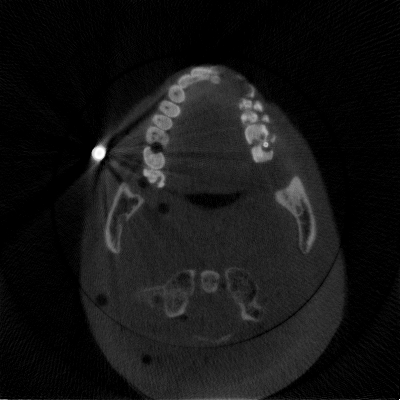

Supplement: S1 Raw images — (ZIP) [file pone.0262713.s002.zip › raw image data PLOS ONE/VMIs/image_VMI_50keV.tif]

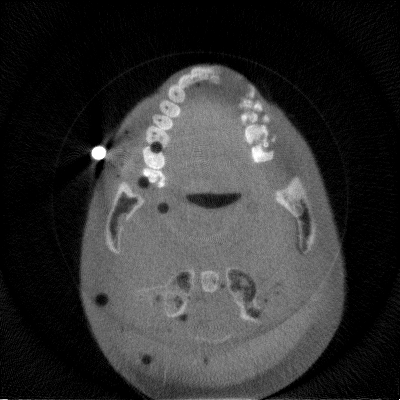

Supplement: S1 Raw images — (ZIP) [file pone.0262713.s002.zip › raw image data PLOS ONE/VMIs/image_VMI_140keV.tif]

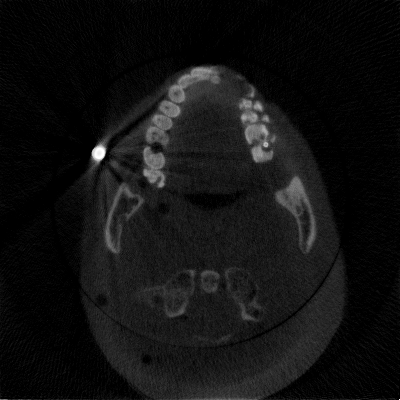

Supplement: S1 Raw images — (ZIP) [file pone.0262713.s002.zip › raw image data PLOS ONE/VMIs/image_VMI_40keV.tif]

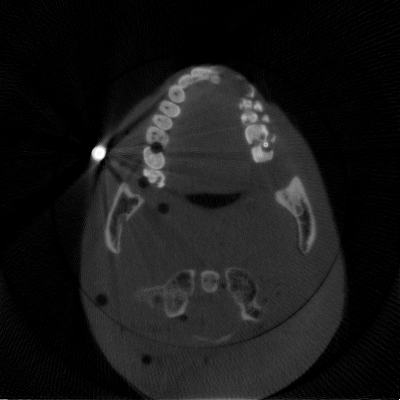

Supplement: S1 Raw images — (ZIP) [file pone.0262713.s002.zip › raw image data PLOS ONE/VMIs/image_VMI_60keV.tif]

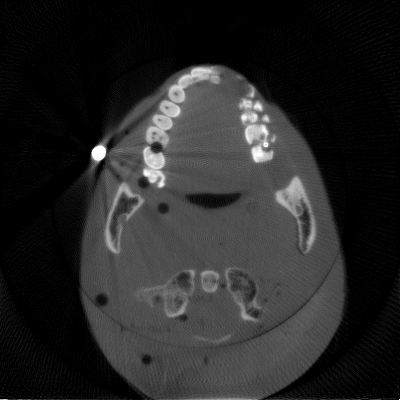

Supplement: S1 Raw images — (ZIP) [file pone.0262713.s002.zip › raw image data PLOS ONE/VMIs/image_VMI_70keV.tif]

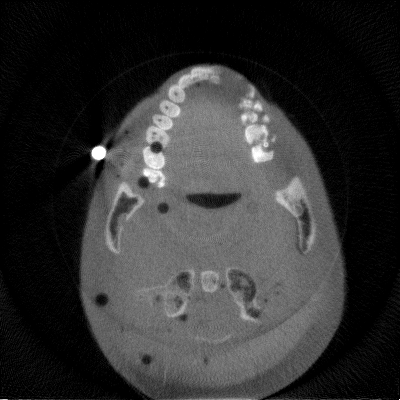

Supplement: S1 Raw images — (ZIP) [file pone.0262713.s002.zip › raw image data PLOS ONE/VMIs/image_VMI_120keV.tif]

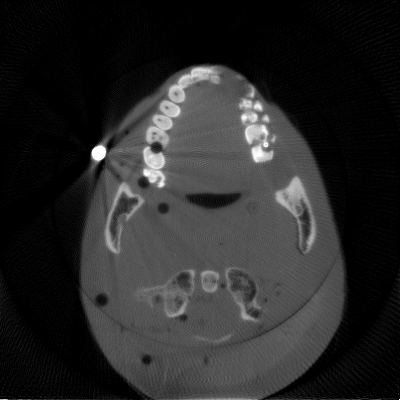

Supplement: S1 Raw images — (ZIP) [file pone.0262713.s002.zip › raw image data PLOS ONE/VMIs/image_VMI_30keV.tif]

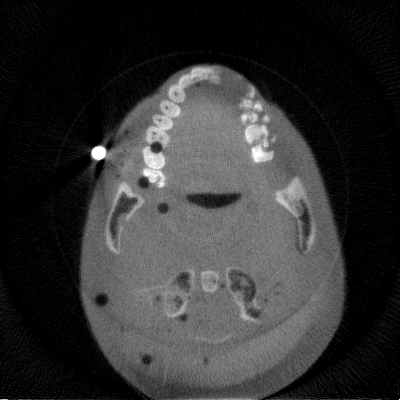

Supplement: S1 Raw images — (ZIP) [file pone.0262713.s002.zip › raw image data PLOS ONE/VMIs/image_VMI_130keV.tif]

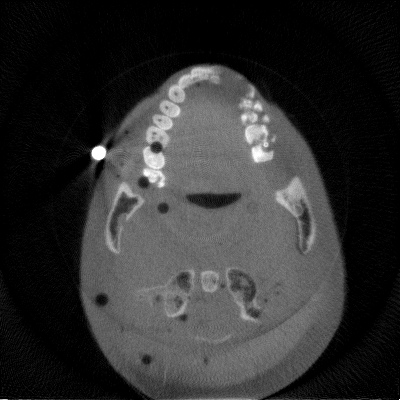

Supplement: S1 Raw images — (ZIP) [file pone.0262713.s002.zip › raw image data PLOS ONE/VMIs/image_VMI_110keV.tif]

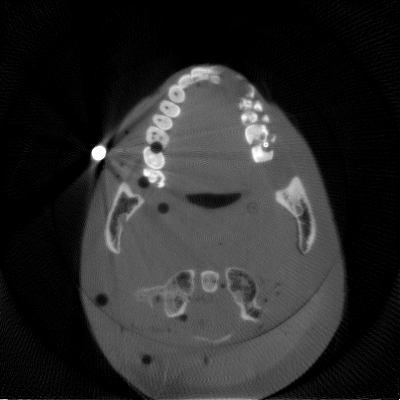

Supplement: S1 Raw images — (ZIP) [file pone.0262713.s002.zip › raw image data PLOS ONE/VMIs/image_VMI_80keV.tif]

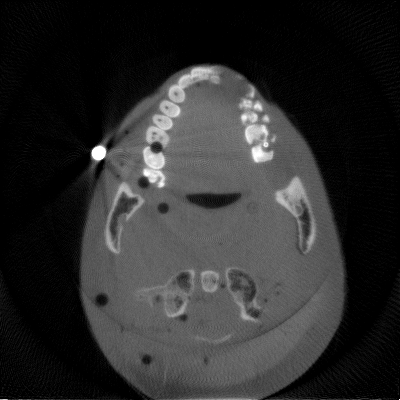

Supplement: S1 Raw images — (ZIP) [file pone.0262713.s002.zip › raw image data PLOS ONE/VMIs/image_VMI_90keV.tif]

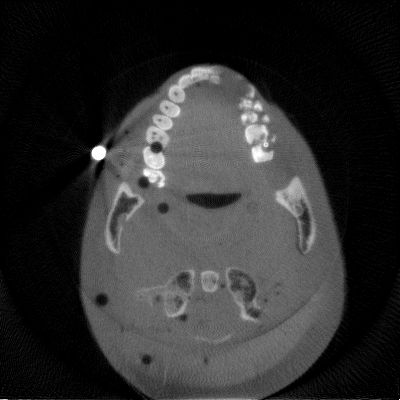

Supplement: S1 Raw images — (ZIP) [file pone.0262713.s002.zip › raw image data PLOS ONE/VMIs/image_VMI_100keV.tif]

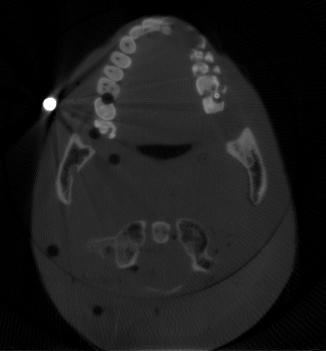

Supplement: S1 Raw images — (ZIP) [file pone.0262713.s002.zip › raw image data PLOS ONE/recons/LE.tif]

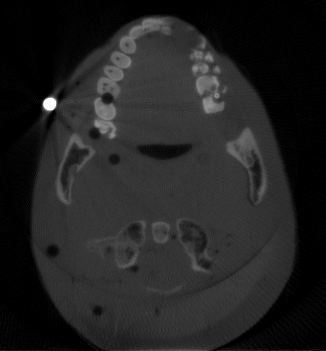

Supplement: S1 Raw images — (ZIP) [file pone.0262713.s002.zip › raw image data PLOS ONE/recons/HE.tif]
